# Model and Methodology to Characterize Phosphor-Based White LED Visible Light Communication Links

**DOI:** 10.3390/s23104637

**Published:** 2023-05-10

**Authors:** Pau Salvador, Vicenç Almenar, Juan Luis Corral, Javier Valls, Maria Jose Canet

**Affiliations:** 1 Instituto de Telecomunicaciones y Aplicaciones Multimedia, Universitat Politècnica de València, 46022 Valencia, Spain; pasallla@upv.es (P.S.); jvalls@upv.es (J.V.); macasu@upv.es (M.J.C.); 2 Instituto Universitario de Tecnología Nanofotónica, Universitat Politècnica de València, 46022 Valencia, Spain; jlcorral@upv.es

**Keywords:** white LED, VLC, link model

## Abstract

LED lighting has become the standard solution for illumination purposes thanks to its energy efficiency. Nowadays, there is growing interest in the use of LEDs for data transmission to develop future-generation communication systems. The low cost and widespread deployment of phosphor-based white LEDs make them the best candidate for visible light communications (VLC), although they have a limited modulation bandwidth. This paper presents a simulation model of a VLC link based on phosphor-based white LEDs and a method to characterize the VLC setup used to perform the data transmission experiments. Specifically, the simulation model incorporates the frequency response of the LED, the noise levels coming from the lighting source and the acquisition electronics, and the attenuation due to both the propagation channel and the angular misalignment between the lighting source and the photoreceiver. In order to validate the suitability of the model for VLC, carrierless amplitude phase (CAP) and orthogonal frequency division multiplexing (OFDM) modulation signals were employed for data transmission, and simulations with the proposed model and measurements over the equivalent scenario show high agreement.

## 1. Introduction

White light LEDs based on phosphor-coated blue LEDs are being massively used for lighting due to their low power consumption and low cost. In recent years, interest has grown in taking advantage of this technology not only for lighting but also for data transmission, thanks to the modulation capacity of LEDs. As a result, visible light communication (VLC) making use of white LEDs is considered an enabling technology for the future generation of communication systems [1].

The main limitation of phosphor-coated white LEDs for data transmission is their small modulation bandwidth: they usually have a −3 dB frequency of a few MHz [2,3]. Therefore, to reach data rates of hundreds of Mb/s, as will be required in future data networks, it is necessary to develop advanced data transmission techniques, such as advanced modulations [4,5,6], multiple access techniques [7,8,9], LED non-linearity compensation [10,11] or the use of multiple LEDs for transmission [12,13]. Any new proposal, after being analytically developed and evaluated, requires validation with experimental measurements in a real VLC setup [14]. During the development stage, it is common to make simplifications or approximations to the real scenario, but it is necessary to verify with actual measurements that these assumptions are valid and do not affect the system performance in real data transmission. Before experimental validation, extensive simulations are usually carried out to develop any new solution [15]; again, at this point, it is common practice to assume simplifications to make this task easier [16]. In both cases, the availability of a realistic simulation model would benefit the development of any new technique because the simulation results would mimic experimental measurements, and then only a final validation with measurements would be required once the simulations are finished. On the other hand, a realistic simulation model can also be beneficial in network planning [17]: the full characterization of the VLC link performance would enable designers to define the number and locations of the access points and estimate the coverage and the achievable data rate at any position before the deployment stage takes place [18].

The electric frequency response of a practical LED must be correctly modeled to obtain accurate data communication performance estimation. The range of phosphor-based white LED models for VLC available in the literature is quite diverse, including first-order low-pass [19], which is combined with a second-order feature to model the yellow component [20], or a second-order one when parasitic components are included [21]. A linear model for white LEDs has recently been proposed [3]. This model separates, on the one hand, the frequency response of the LED and its fixture and, on the other hand, the response of the phosphor coating. The parameter extraction for this model is based on a series of frequency response measurements and the use of blue and yellow filters.

The main contributions of this work are (1) a simulation model of a real VLC link that considers the frequency response of the white LED, the radiation pattern of the lighting fixture, the noise levels and the optical channel propagation; and (2) a method to characterize the frequency response and the different noise terms of the VLC system used to carry out the experiments.

The rest of the paper has the following structure. Section 2 introduces the modulations used to verify the simulation model. Section 3 presents the linear model used to characterize the white LEDs. Section 4 details the VLC link model. Section 5 shows the experimental setup and exposes the method to calibrate the signal and noise of the model according to the measurements. Section 6 exposes the procedure to perform the VLC link simulation. Section 7 presents the measurements performed to validate the model. Finally, the conclusions are stated in Section 8.

## 2. Modulation Schemes

Carrierless amplitude phase (CAP) modulation and orthogonal frequency division multiplexing (OFDM) are modulation schemes usually employed in VLC research [22,23,24,25]. Therefore, both are used in this paper to show the suitability of the proposed methodology to characterize a VLC link. As the signal modulates the intensity of the light and it cannot be negative, both modulation schemes require the addition of a DC bias to the signal to make it positive. In most VLC scenarios, the LEDs at the transmitter will be simultaneously used for lighting so their DC bias will provide the bias required by the modulation signals.

### 2.1. CAP

CAP modulation was developed during the 1990s as a transmission scheme for very high-speed digital subscriber lines (VDSL) over copper wires [26]. CAP is a different implementation of quadrature amplitude modulation (QAM) where a band-pass pulse shaping filter substitutes the modulation stage with a carrier. This alternative is computationally efficient when the baseband signal is shifted to low frequencies in the order of the symbol rate [27]. If $a_{k}$ and $b_{k}$ are the in-phase and quadrature components of a QAM symbol, the CAP-transmitted signal waveform of *L* symbols is

(1) $$v_{s}\left(t\right)=\sum_{k=0}^{L-1}\left(a_{k}\;g_{I}\left(t-kT\right)+b_{k}\;g_{Q}\left(t-kT\right)\right),$$

where *T* is the symbol period and $g_{I}\left(t\right)$ and $g_{Q}\left(t\right)$ are the in-phase and quadrature band-pass pulse shaping filters, which can be generated using a root-raised cosine (RRC) filter p(t) modulating a cosine/sine at the carrier frequency $f_{c}$ used for transmission: $g_{I}\left(t\right)=p\left(t\right)\cdot \cos\left(2\pi f_{c}t\right)$, $g_{Q}\left(t\right)=p\left(t\right)\cdot \sin\left(2\pi f_{c}t\right)$.

Given the bandwidth occupied by CAP, $BW_{\mathrm{CAP}}=\left(1+\beta \right)/T$, where β is the excess bandwidth (also known as roll-off) of the RRC filter, and the low-pass nature of the LED bandwidth, it is common to employ a band near DC with

(2) $$f_{c}=\frac{1+\beta }{2T}$$

At the receiver, data symbols are extracted from the received signal by making use of a matched filter for each branch. Both matched filters are the same RRC filters (modulated by a cosine/sine) used at the transmitter. The in-phase and quadrature filter outputs are combined to obtain the complex QAM symbol. When high data rates are required, the CAP signal has a bandwidth larger than the LED 3 dB modulation bandwidth. As a result, the CAP signal is distorted and the receiver requires the use of an equalizer to recover the transmitted bits [28].

### 2.2. OFDM

Alternatively, given the low-pass characteristic of the VLC channel, instead of using a single carrier approach, multicarrier modulation such as OFDM can be employed to avoid the need for complex equalizers. Moreover, the number of transmitted subcarriers and the number of bits assigned to each one can be adapted to the frequency response of the LED to maximize the data throughput.

A block of $N_{\mathrm{fft}}$ OFDM signal samples x[n] (with $0\leq n<N_{\mathrm{fft}}$) are generated by an $N_{\mathrm{fft}}$-point inverse fast Fourier transform (FFT) following [29]:

(3) $$x\left[n\right]=\frac{1}{N_{\mathrm{fft}}}\sum_{q=0}^{N-1}X_{q}\;\exp\left(\frac{j2\pi nq}{N_{\mathrm{fft}}}\right),$$

where $X_{q}$ is the complex QAM symbol to be sent in the *q*-th subcarrier. As the generated signal x[n] must be real, $X_{q}$ must fulfill Hermitian symmetry:

(4) $$X_{q}=X_{N_{\mathrm{fft}}-q}^{*}\;\;\;\mathrm{for}\;\;\;0<q<\frac{N_{\mathrm{fft}}}{2}$$

and components $X_{0}$ and $X_{N_{\mathrm{fft}}/2}$ are set to zero.

To cope with the delay spread of the VLC channel, the last $N_{\mathrm{cp}}$ samples of x[n] are pre-pended to x[n] to create an OFDM symbol with a length of $N_{\mathrm{cp}}+N_{\mathrm{fft}}$ samples; this procedure is usually known as cyclic prefix. Finally, the OFDM signal, $v_{s}\left[n\right]$, will be created by concatenating all the obtained OFDM symbols. After digital to analog conversion (DAC), the output signal $v_{s}\left(t\right)$ will be generated.

The generated signal $v_{s}\left(t\right)$ has a bandwidth that depends on the number of employed subcarriers ($N_{\mathrm{sc}}$) and the sampling frequency ($F_{s}$) of the DAC according to $BW_{\mathrm{OFDM}}=N_{\mathrm{sc}}\cdot F_{s}/N_{\mathrm{fft}}$.

## 3. White LED Model

**Figure 1:**
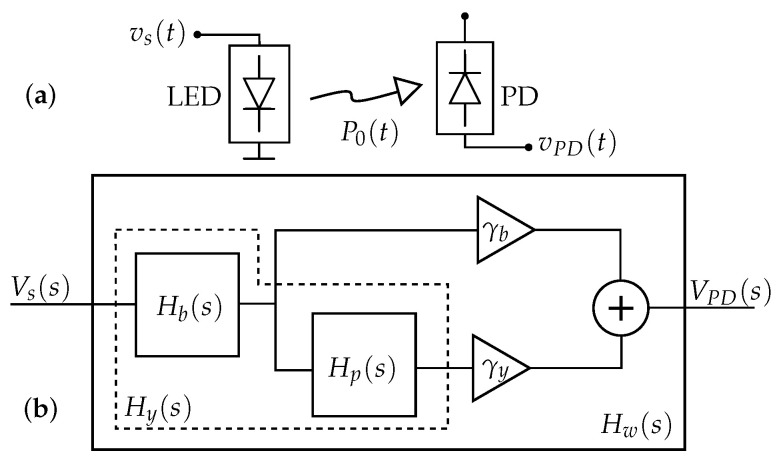
(**a**) System to be modeled. (**b**) Block diagram of the white LED model.

In [3], a linear system model for the phosphor-coated white LED was proposed (Figure 1b), which separates the effect of the blue LED (electrical domain) and phosphor coating (optical domain). The model assumes that the optical power generated by the LED, $P_{0}\left(t\right)$, due to its modulating voltage, $v_{s}\left(t\right)$, is detected by a photodetector (PD) whose output voltage ($v_{PD}\left(t\right)$) is proportional to this optical power, as shown in Figure 1a. Additionally, all DC or bias terms at the LED or PD are not considered as the model only takes into account the data signals to be transmitted.

The blue LED is modeled as a 2nd-order system (Equation (5)) where $-p_{b1}$ and $-p_{b2}$ are two real poles and $k_{b}=H_{b}\left(0\right)$.

(5) $$H_{b}\left(s\right)=\frac{k_{b}}{\left(\frac{s}{p_{b1}}+1\right)\left(\frac{s}{p_{b2}}+1\right)}$$

The phosphor coating is modeled as a 1st-order system (Equation (6)) where $-p_{p}$ is a real pole and $k_{p}=H_{p}\left(0\right)$.

(6) $$H_{p}\left(s\right)=\frac{k_{p}}{\frac{s}{p_{p}}+1}$$

The model of the generated yellow wave is obtained as the cascade of the blue LED and the effect of the phosphor transfer functions, as in Equation (7).

(7) $$H_{y}\left(s\right)=H_{b}\left(s\right)\cdot H_{p}\left(s\right)$$

Figure 1b shows the white LED complete model and it is formulated in Equation (8), where $\gamma_{b}$ and $\gamma_{y}\cdot k_{p}$ are the relative weights with which the blue and yellow components are added at the receiver, respectively.

(8) $$H_{w}\left(s\right)=\gamma_{b}\cdot H_{b}\left(s\right)+\gamma_{y}\cdot H_{y}\left(s\right)$$

Finally, the transfer function $H_{w}\left(s\right)$ can be rewritten as

(9) $$H_{w}\left(s\right)=\frac{k_{T}\left(\frac{s}{z_{p}}+1\right)}{\left(\frac{s}{p_{b1}}+1\right)\left(\frac{s}{p_{b2}}+1\right)\left(\frac{s}{p_{p}}+1\right)},$$

where $k_{T}\;=\;k_{b}\;\gamma_{b}\left(1+\frac{k_{p}\gamma_{y}}{\gamma_{b}}\right)$ and $z_{p}\;=\;p_{p}\left(1+\frac{k_{p}\gamma_{y}}{\gamma_{b}}\right)$. When necessary, the time domain impulse response of the system $h_{w}\left(t\right)$ can be obtained from the Laplace transfer function $H_{w}\left(s\right)$.

## 4. VLC Link Model

**Figure 2:**
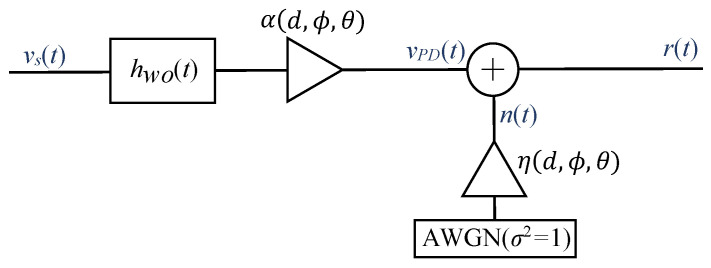
Block diagram of the VLC link model.

The VLC channel includes the driver, the LED, the optical channel, the photodiode, and the trans-impedance amplifier (TIA). The LED frequency response is generally assumed to be the most restrictive bandwidth limitation of the VLC link. Therefore, the white LED model from Section 3 can accurately describe the frequency response of the VLC link if the LED driver and the TIA are designed not to change the LED frequency response. The VLC channel can be modeled as a baseband linear system with a transmitted signal (CAP or OFDM) $v_{s}\left(t\right)$ over a channel impulse response $h_{w}\left(t\right)$ with additive white Gaussian noise (AWGN) n(t). Thus, the received signal r(t) can be expressed as

(10) $$r\left(t\right)=v_{PD}\left(t\right)+n\left(t\right)=v_{s}\left(t\right)⊗h_{w}\left(t\right)+n\left(t\right),$$

where $h_{w}\left(t\right)=\;h_{wo}\left(t\right)\cdot \alpha \left(d,\phi ,\theta \right)$ and $n\left(t\right)=\;n_{o}\left(t\right)\cdot \eta \left(d,\phi ,\theta \right)$, being $h_{wo}\left(t\right)$ the channel impulse response measured at a given distance (with the LED and PD perfectly aligned), $n_{o}\left(t\right)$ a unitary variance AWGN, and α(d,ϕ,θ) is an attenuation parameter that depends on the distance between the LED and PD and their relative alignment. Finally, η(d,ϕ,θ) is the noise standard deviation at the receiver that depends on the distance between the LED and PD and their relative alignment due to the shot noise component, as will be shown in Section 4.2. Figure 2 shows the block diagram of the VLC link model.

The parameters of the white LED model described in Section 3 can be easily extracted for any given configuration of the VLC channel (fixed optical channel length and transmitter and receiver angular alignment) by following the procedure described in [3]. However, if we are interested in modeling the system for different distances or angular positions, the parameters should be extracted several times. With the model and methodology proposed in this paper, this problem can be avoided, and, with only a few measurements, the link can be modeled for different distances and alignments.

### 4.1. Propagation Model

**Figure 3:**
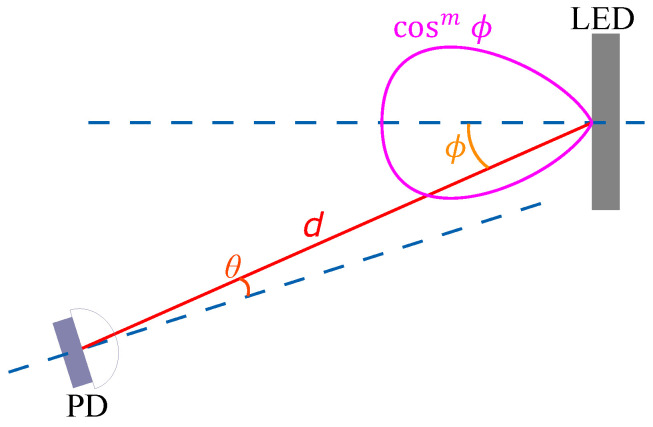
Generic VLC scenario with angular misalignment between LED and photoreceiver.

The optical channel is usually modeled in terms of optical power as [30]

(11) $$\frac{P_{PD}}{P_{LED}}=\frac{\left(m+1\right)AJ\left(\phi \right)\cos^{m}\left(\phi \right)}{2\pi d^{2}}G\left(\theta \right)\cos\left(\theta \right),$$

where *d* is the length of the optical channel, $P_{LED}$ is the optical power transmitted by the LED, $P_{PD}$ is the optical power detected by the photodiode, *A* is the surface area of the PD, ϕ is the transmitting angle related to the angle of maximum radiation of the LED, and θ is the receiving angle at the PD related to the normal of the PD surface, as depicted in Figure 3. The LED radiation pattern is modeled with an *m*-th order generalized Lambertian pattern with axial symmetry: $\cos^{m}\left(\phi \right)$. The term J(ϕ) in Equation (11) is an optional term to model any possible deviation from the generalized Lambertian pattern of LED radiation due to the insertion of a reflector. Finally, G(θ) is the combined angular pattern of the gain of the lens, the concentrator, and the optical filter that are optionally used at the receiver.

Equation (11) allows us to use the LED model for different VLC scenarios without extracting its parameters again. For instance, if we have characterized the VLC channel, $h_{wo}\left(t\right)$, for a distance $d=d_{0}$ and angles ϕ=0 and θ=0, the channel impulse response for other distances, $h_{w}\left(t\right)$, or any transmitter/receiver alignment can be expressed as

(12) $$h_{w}\left(t\right)=h_{w0}\left(t\right)\alpha \left(d,\phi ,\theta \right)=h_{w0}\left(t\right)\bar{J(\phi )}\cos^{m}\left(\phi \right)\bar{G(\theta )}\cos\left(\theta \right){\left(\frac{d_{0}}{d}\right)}^{2},$$

where $\bar{J(\phi )}$ and $\bar{G(\theta )}$ are J(ϕ) and G(θ) normalized to J(0) and G(0), respectively.

### 4.2. Noise Sources

Concerning the noise component in Equation (10), the total noise measured at the output of the receiver will be a combination of independent noise terms, which can be grouped into two categories according to their dependence on the level of the incident optical power at the photodetector.

- The detected-power independent noise term, $N_{rx}$, covers the thermal noises of the photodiode and the TIA, the dark current noise of the photodiode, and the thermal and quantization noise of the data acquisition equipment. This noise is usually modeled as an additive noise signal with Gaussian distribution.
- The detected-power dependent noise term, $N_{sh}$, is the shot noise of the photodiode, whose power is known to be proportional to the optical incident power. Shot noise follows a Poisson distribution that can be approximated to a Gaussian distribution for high optical incident power, as is usually the case in a typical VLC scenario.

Considering these categories, the noise component in Equation (10) follows a Gaussian distribution with a power equal to the sum of the powers of both types of noises, $N=N_{rx}+N_{sh}$. Suppose that we are able to characterize the receiver noise, $N_{rx}$, and the shot noise, $N_{sho}$, for the same reference VLC channel ($d_{0}$, $\phi_{o}=0$ and $\theta_{o}=0$), as in Section 4.1. In this case, the noise power, *N*, of the noise signal in Equation (10) can be calculated for any distance or alignment angle as

(13) $$N=N_{rx}+N_{sh}=N_{rx}+N_{sho}\bar{J(\phi )}\cos^{m}\left(\phi \right)\bar{G(\theta )}\cos\left(\theta \right){\left(\frac{d_{0}}{d}\right)}^{2}.$$

## 5. Measurement Setup and Model Calibration

In order to assess the performance of an actual VLC data link, we need to obtain the LED frequency response, the noise power levels, and the angular pattern of both the LED and the receiver. This section describes the experimental setup required to calibrate the simulation model.

The model characterization is carried out with a reference distance between the LED and PD equal to $d_{0}=130$ cm and perfect alignment (θ=0, ϕ=0) between them except when the angular patterns of the LED and PD are measured. The simulation VLC model will be used in Section 7 to assess the performance of the VLC link at other distances or angular orientations.

### 5.1. Measurement Setup

**Figure 4:**
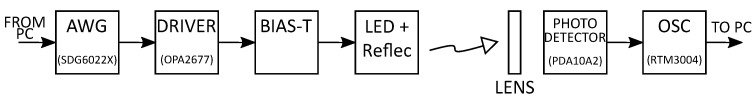
Scheme of the white LED setup.

**Figure 5:**
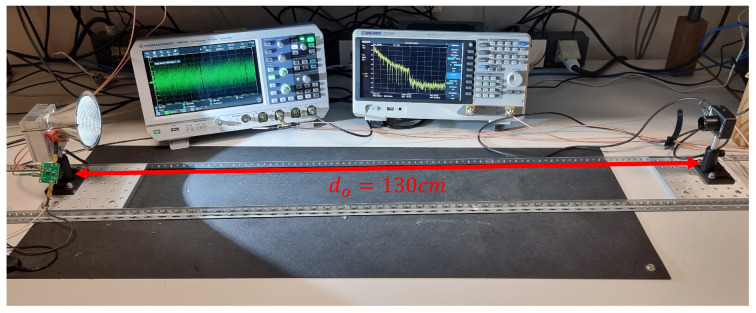
Setup used to calibrate the VLC link. LED and PD are at a distance $d_{0}\;=\;130$ cm and aligned (ϕ=0° and θ=0°).

Figure 4 shows the measurement setup block diagram and Figure 5 its implementation, used to calibrate the model. The transmitter consists of an arbitrary wave generator (AWG), Siglent SDG6022X, a driver based on bias-T with a capacity of 22 µF and an inductance of 1 mH and low output impedance implemented with an OPA2677 [31], which are connected to the LED under test with a reflector C16902_ALISE-110-WW. At the receiver, the lens ACL25416U-A is used with the PDA10A2 amplified photodetector, whose output is connected to the Rohde&Schwarz RTM3004 oscilloscope (OSC). Both the AWG and OSC are controlled from MATLAB to generate and capture the transmitted and received signal, respectively. Relative to the PDA10A2, its small signal bandwidth is 150 MHz, trans-impedance gain $5\cdot 10^{3}$ V/A, and noise equivalent power (NEP) $2.92\cdot 10^{-11}$ W/$\sqrt{\mathrm{Hz}}$.

### 5.2. White LED Characterization

Two different white LEDs were characterized:

- LED1: CXB1830-0000-000N0BV265E from Cree;
- LED2: LZ4-40CW08-0065 from OSRAM.

Their main features and the bias current used in the experiments are summarized in Table 1. The two devices exhibit high luminous flux. LED1 is a COB (chip-on-board) diode type, and LED2 is composed of 4 LEDs connected in series.

**Figure 6:**
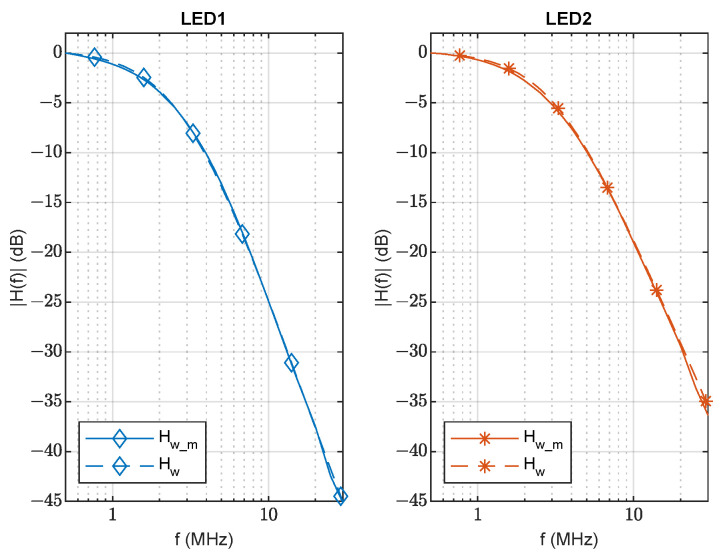
Comparison between measured ($H_{w\_m}$) and estimated ($H_{w}$) frequency responses of LED1 and LED2.

The parameters of LED1 and LED2 were obtained using the method described in [3] for a reference VLC channel with $d_{0}\;=\;130$ cm and perfect alignment between the LED and photodetector (θ=0, ϕ=0). The estimated parameters are indicated in Table 2, and their frequency responses are shown in Figure 6, where solid lines correspond to the measured frequency responses of LED1 and LED2, and dashed lines were obtained using Equation (9) with the extracted parameters. During measurements, the LEDs were biased with the corresponding $I_{bias}$ indicated in Table 1, and the modulating signal ($v_{s}$) amplitude was set to 900 mV_pp_ to keep the signal in the linear zone.

### 5.3. LED and Photodetector Angular Characterization

To characterize the radiation pattern of each LED, the order of the Lambertian pattern, *m*, can be estimated by rotating the LED and measuring the detected voltage (proportional to the detected optical power) at two different angles, $\phi_{1}=20$ and $\phi_{2}=40$ degrees in our case. These measurements were made at the reference distance $d_{0}=130$ cm. Orders $m_{1}=2.29$ and $m_{2}=2.88$ were calculated for both LEDs under study using the following expression:

(14) $$m=\frac{\log_{10}\left(|V_{PD1}\left|/\right|V_{PD2}|\right)}{\log_{10}\left(\cos\left(\phi_{1}\right)/\cos\left(\phi_{2}\right)\right)}$$

**Figure 7:**
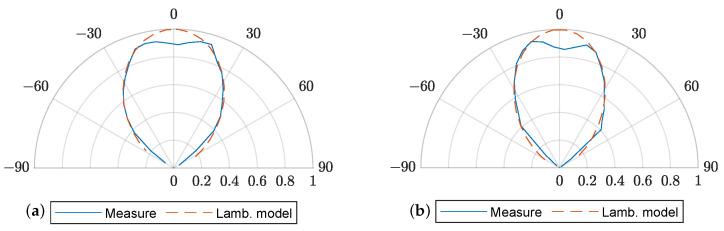
Combined radiation pattern of the LED and the reflector (measurements in blue, generalized Lambertian model in dashed orange). (**a**) LED1, (**b**) LED2.

The particular reflector used in combination with the LEDs under test, C16902_ALISE-110-WW, causes a notch in the radiation pattern around ϕ=0, as depicted in Figure 7 for both LED1 and LED2. This deviation from the generalized Lambertian model was taken into account to obtain J(ϕ) for ϕ from −15 to 15 degrees.

The angular sensitivity of the PDA10A2 photodetector with the aspheric lens placed in front of it was shown to be very high. Thus, almost perfect alignment between the LED and PD is required, which means $\bar{G(\theta \approx 0)}=1$. On the contrary, $\bar{G(\theta \neq 0)}=0$ when the LED and the PD are not properly aligned.

### 5.4. Noise Level Calibration

The receiver and acquisition noise ($N_{rx}$) was measured at the detector output with the photodetector turned on but without any incident light. This measurement was done with the same acquisition system used for all the data measurements. The value obtained was $N_{rx}=750.51$ nW.

Next, the LED was turned on and biased with $I_{bias}$, as shown in Table 1. The noise level at the PD output with normal lighting conditions (*N*) was measured, with the PD placed at the reference distance $d_{0}=130$ cm and aligned with the LED. Then, the shot noise level for the reference distance ($N_{sho}$) could be obtained by subtracting the previously measured thermal and acquisition noise ($N_{rx}$) from *N*:

(15) $$N_{sho}=N-N_{rx}$$

The corresponding noise power levels were $N_{sho}=12.48$ μW (LED1) and $N_{sho}=2.91$ μW (LED2). Once $N_{rx}$ and $N_{sho}$ are measured, Equation (13) can be used to obtain the noise power levels to be included in the simulations for any distance or angular misalignment between an LED and PD.

## 6. Simulation Procedure

Next, the sequence of steps to perform a simulation of the VLC link shown in Figure 2 is described.

1. Generate the discrete-time sequence to be transmitted by the LED, $v_{s}\left[n\right]$:
  - Generate the CAP/OFDM sequence using a sampling frequency of $f_{s}\;=\;100$ MHz ($T_{s}\;=\;10$ ns) and an amplitude of 900 ${\mathrm{mV}}_{\mathrm{pp}}$.
  - Simulate the effect of the DAC used by the arbitrary wave generator: signal $v_{tx}\left[n\right]$ is quantized with a 16-bit resolution and filtered with a sinc$\left(f/f_{s}\right)$ shape frequency response. As a result, we obtain the signal $v_{s}\left[n\right]$.
2. Filter $v_{s}\left[n\right]$ by the white LED linear model to obtain $v_{PD}\left[n\right]$:
  - Convert the LED linear model specified in Equation (9) from continuous to discrete time by obtaining the equivalent z-transform response, $H_{w}\left(z\right)$. For example, we made use of the least-squares method with $T_{s}\;=\;10$ ns provided by the c2d MATLAB function.
  - Filter $v_{s}\left[n\right]$ with $H_{w}\left(z\right)$.
  - Scale the filtered output according to distance (*d*) and alignment angles (θ, ϕ) by multiplying with α(d,ϕ,θ) from Equation (12).
3. Add noise to the detected signal, $v_{PD}\left[n\right]$:
  - Calculate the total noise variance (*N*) according to the distance (*d*) and alignment angles (θ, ϕ).
  - Generate an AWGN sequence with $T_{s}=10$ ns and variance *N* over 50 Ω:
    (16) $$\eta \left(d,\phi ,\theta \right)=\sqrt{50N}=\sqrt{50\left(N_{rx}+N_{sh}\right)}$$
  - Add signal and noise sequences to obtain r[n].
4. Make use of a CAP/OFDM receiver to obtain the detected bits after transmission.

## 7. VLC Data Link Evaluation

This section shows measurements performed with the setup of Section 5.1 (Figure 5) and simulations (following the procedure described in the previous section), which are compared to validate the VLC link model. First, CAP and OFDM modulations were evaluated at a fixed distance $d_{0}$ in Section 7.1 and Section 7.2, respectively. Second, the model calibrated at $d_{0}$ was employed in Section 7.3 to characterize the throughput obtained using OFDM at different distances and angles with a more realistic VLC setup.

### 7.1. Measurements and Simulations with CAP Modulation at $d_{0}\;=\;130$ cm, ϕ=0° and θ=0°

**Figure 8:**
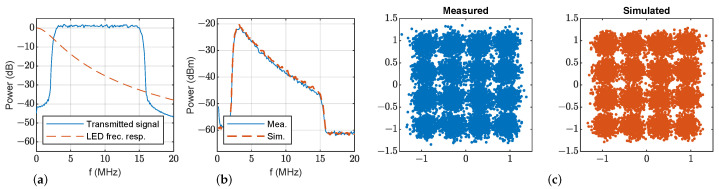
16-QAM CAP modulation transmission with LED1 at $d_{0}\;=\;130$ cm, ϕ=0° and θ=0°: (**a**) transmitted power spectrum and estimated frequency response; (**b**) measured (blue) and simulated (dashed orange) power spectrum of the received signal; (**c**) measured (blue) and simulated (orange) scatter diagram of the demodulated signal.

**Figure 9:**
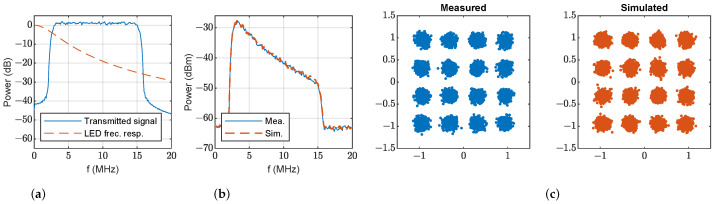
16-QAM CAP modulation transmission with LED2 at $d_{0}\;=\;130$ cm, ϕ=0° and θ=0°: (**a**) transmitted power spectrum and estimated frequency response, and (**b**) measured (blue) and simulated (dashed orange) power spectrum of the received signal; (**c**) measured (blue) and simulated (orange) scatter diagram of the demodulated signal.

In the first set of trials, a CAP modulation was employed to transmit 16-QAM data symbols. The RRC filter roll-off factor was set to 0.1 and the symbol rate to 12.5 MHz. This gives a modulated signal with a bandwidth of 13.75 MHz, centered at 9 MHz with a throughput of 50 Mb/s. Figure 8 and Figure 9 show, for both LED1 and LED2, (a) the transmitted 16-QAM CAP signal power spectrum and the estimated frequency response of the LED; (b) the measured power spectrum of the received CAP signal after photodetection and its estimation; and (c) the simulated and measured constellation diagram of the demodulated signal. As can be seen, the VLC link frequency response is accurately modeled, and the proposed method scales properly the noise floor: simulation and measurement noise overlap in the frequency response plots and symbol constellations have the same dispersion.

After the demodulation of the data symbols, the channel distortion caused by the LED needs to be compensated for, which is solved using a linear equalizer working at the symbol rate. The order of the equalizer was fixed to 60 symbols in order to fairly compare measurements, simulations, and LEDs. Figure 8c and Figure 9c show the measured and simulated scatter diagrams of the demodulated and equalized signals for both LEDs, and Table 3 contains the error vector magnitude (EVM), showing that simulations with the proposed model match the measurements. On the other hand, the low EVM values given by LED2 show that its performance is clearly superior because of its wider bandwidth, which introduces lower distortion in the transmitted CAP signals. The obtained bit error rate (BER) for LED1 is 5.9 × $10^{-3}$; meanwhile, for LED2, the BER is 0.

### 7.2. Measurements and Simulations with OFDM at d=130 cm, ϕ=0° and θ=0°

**Figure 10:**
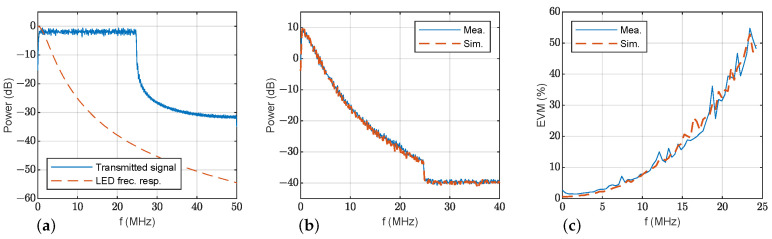
OFDM transmission at $d_{0}\;=\;130$ cm, ϕ=0° and θ=0°: (**a**) transmitted power spectrum (solid blue) and estimated frequency response of LED1 (dashed orange); (**b**) measured (solid blue) and simulated (dashed orange) power spectrum of the received signal with LED1; (**c**) measured (solid blue) and simulated (dashed orange) EVM of the received signal.

**Figure 11:**
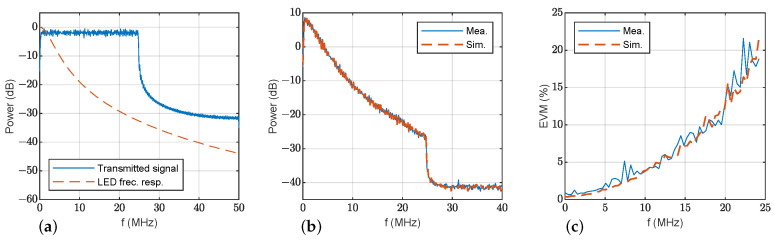
OFDM transmission at $d_{0}\;=\;130$ cm, ϕ=0° and θ=0°: (**a**) transmitted power spectrum (solid blue) and estimated frequency response of LED2 (dashed orange); (**b**) measured (solid blue) and simulated (dashed orange) power spectrum of the received signal with LED2; (**c**) measured (solid blue) and simulated (dashed orange) EVM of the received signal.

This section presents results using OFDM modulation to show the merits of the proposed simulation model. Table 4 contains the parameters employed to configure the OFDM data transmission. Figure 10a shows the transmitted OFDM signal power spectrum, and the estimated frequency response of LED1 using the proposed model. Power spectra of signals and noise at the receiver are presented in Figure 10b: measurements are shown in solid blue lines and simulations with the proposed model in dashed orange line. Again, the similarity between the measurements and simulations with the proposed model both for signal and noise is shown. The estimated EVM for each OFDM subcarrier is displayed in Figure 10c. For the sake of completeness, Figure 11 represents the same results for LED2. These results are consistent with the previous discussion: the simulations and measurements agree. Again, the EVM performance of LED2 is clearly superior to that of LED1 thanks to its wider bandwidth.

As shown above, the proposed model accurately estimates the performance of the VLC data link. An interesting application of this model is to use the EVM estimation to adjust the adaptive bit loading of the OFDM subcarriers [32] to maximize the achievable bit rate of the VLC link. The adaptive bit loading can be implemented as follows. Consider that the LED frequency response has been previously characterized, so $H_{w}\left(s\right)$ is known. The EVM is estimated for a particular link configuration (*d*, ϕ and θ) by simulating the VLC system with OFDM using 4-QAM modulation in all subcarriers. Then, the SNR of each subcarrier can be obtained using the estimated EVM [33]. Taking into account that a data link admits a maximum BER, the SNR is employed to select the QAM order of each subcarrier that keeps the BER below this value. In this case, the total throughput is calculated by aggregating the number of bits transmitted by all the subcarriers. In the measurements carried out in this paper, the objective was to obtain a BER under 3.8 × $10^{-3}$, which allows for error-free transmission if forward error correction (FEC) with hard detection (HD) is employed [34] (also known as the HD-FEC threshold in the literature). Table 5 shows the modulation order (*M*-QAM) used at each subcarrier; following this bit loading strategy, LED1 achieves a throughput of 84.77 Mb/s, and LED2 of 176.95 Mb/s.

### 7.3. Characterization of a VLC Cell

**Figure 12:**
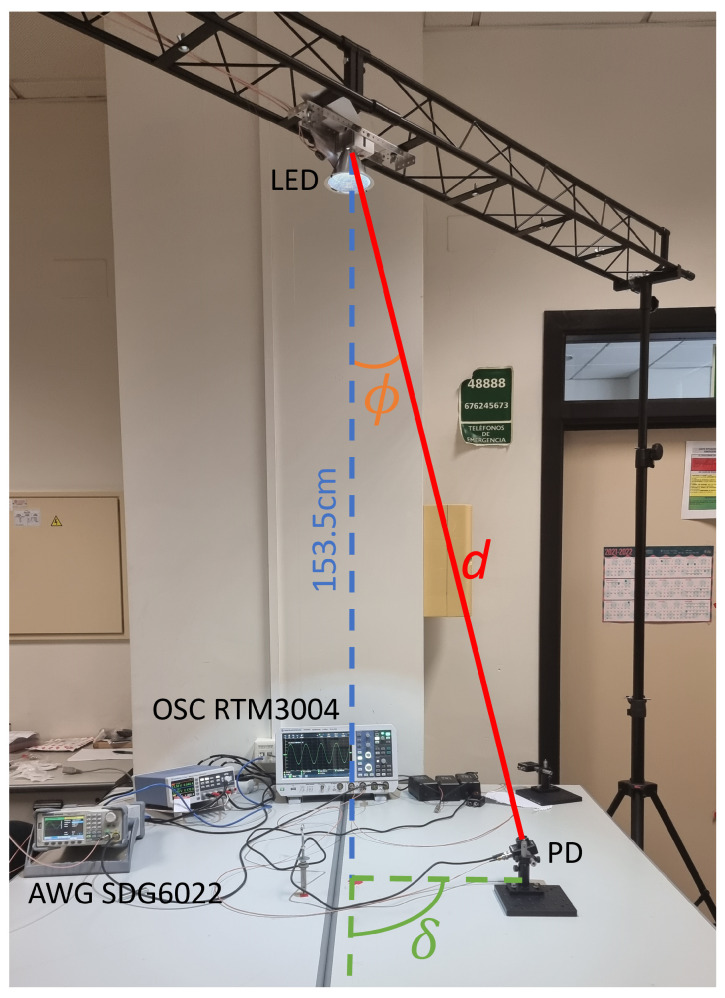
Setup used to characterize a VLC link. LED and PD are at an arbitrary distance *d* and angle ϕ (with θ=0°).

In this section, we make use of our simulation model to characterize the throughput performance of a VLC cell using OFDM modulation with LED2. Figure 12 shows the experimental VLC cell, where the LED used for transmission and illumination is located over two office tables 153.5 cm below the LED. A PD is located on this surface and its position with respect to the LED is determined using angles ϕ (defined in Section 4) and δ (which is used here to define the angle rotation in the horizontal plane of the PD position around the LED), both shown in Figure 12. The PD is always aligned to the LED; therefore, we can consider θ=0.

**Figure 13:**
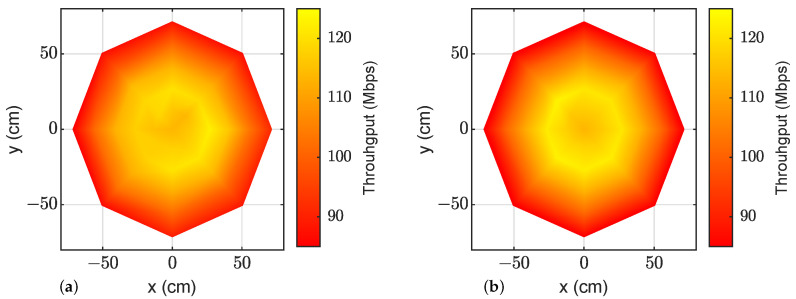
OFDM transmission coverage colormap with LED2: (**a**) represents measurements using the VLC cell setup; (**b**) represents the estimated values using the proposed methodology.

The procedure described in the previous section was followed to estimate the throughput at each evaluated position. Once the adaptive bit loading was calculated, this configuration was employed in data transmission measurements to check the validity of the VLC link evaluation procedure. Table 6 shows the obtained values and Figure 13 represents the same information using a colormap: onsite measurements are shown on the left and estimations on the right. Both the table and figure show the agreement between the measurements and estimations along the cell surface, proving the validity of the proposed methodology. When comparing each pair of simulated and measured data in Table 6, an RMS error equal to 2.65 Mb/s (or 2.52% in relative terms) can be calculated for the whole VLC cell.

The previous OFDM measurements and simulations were carried out under ideal conditions, without considering any environmental disturbance. In an indoor link, assuming that there is no light blockage at the PD, there are two possible sources of environmental disturbance: (1) lighting changes in the room caused by external light (i.e., sunlight from windows); and (2) ambient light from fluorescent tubes used in combination with LEDs to provide illumination. For the first source, we measured the increase in shot noise due to sunlight by placing the PD focused on a window (but not receiving direct sunlight). With this increase in noise, the performance of the VLC link was simulated and a throughput deterioration arose: from 122.4 to 115.3 Mbps (6%) at d=155.9 cm and ϕ=10°, and from 91.2 to 80.9 Mbps (11%) at d=169.4 cm and ϕ=25°. For the second source, we verified that the interference from the fluorescent tubes did not affect the OFDM signal because its maximum frequency was lower than the first subcarrier used for data transmission (390.625 kHz).

Finally, measurements and simulations were performed at different distances and angles, transmitting CAP signals while keeping LED2 on the transmitter. They were generated with a symbol rate of 12.5 MHz, an RRC filter roll-off factor 0.1, and a central frequency of 9 MHz. The obtained EVM and throughput values are shown in Table 7. A modulation order of 128-QAM can be used if EVM is lower than 6% to maintain the BER below the HD-FEC threshold, whereas 64-QAM is required for EVM between 6% and 8.5%. Again, the proposed simulation model is shown to emulate the real behavior of a VLC transmission for CAP signals.

## 8. Conclusions

This article presents a model and methodology to simulate a complete VLC link based on phosphor-coated white LEDs. The model includes the frequency response of the LED, which is the most restrictive element of the transmission chain, the radiation pattern of the combination of the LED and reflector, the distance and angle between the LED and the photodetector, and, finally, the noise introduced into the VLC link by the receiver electronics and the incident light. The model only needs to be calibrated at a given set of distances and angles between the LED and the photodetector. Once calibrated, simulations can be performed at arbitrary distances and angles. The model was validated by simulations and measurements using OFDM and CAP modulations. In addition, the paper also presents a procedure to adjust the bit loading of an OFDM modulation using simulations carried out with the model. Finally, the model was employed to estimate the throughput variations inside a VLC cell and a small difference (2.52% average error across the whole cell) was found between the simulation data and actual throughput measurements.

## Figures and Tables

**Table 1:** LEDs features and $I_{bias}$.

Feature	LED1	LED2
Correlated Color Temperature	6500	6500
Forward Voltage (V)	35	12.6
DC Forward Current (mA)	800	700
Luminous Flux (lm)	4600	800
Viewing Angle (deg.)	115	90
$I_{bias}$ (mA)	400	650

**Table 2:** Estimated LED parameters.

Parameters	LED1	LED2
$k_{b}$	$6\cdot 10^{-2}$	$6.0\cdot 10^{-2}$
$p_{b1}\left(s^{-1}\right)$	$1.45\cdot 10^{7}$	$2.01\cdot 10^{7}$
$p_{b2}\left(s^{-1}\right)$	$5.91\cdot 10^{7}$	$12.56\cdot 10^{7}$
$k_{p}$	11.5	8.2
$p_{p}\left(s^{-1}\right)$	$1.95\cdot 10^{7}$	$2.20\cdot 10^{7}$
$\gamma_{b}$	3.4	3
$\gamma_{y}$	1.09	1.05

**Table 3:** Measured and simulated EVM (%) of the received CAP signal.

LED	Measured	Simulated
LED1	17.4	17.6
LED2	2.8	2.6

**Table 4:** OFDM transmission parameters.

QAM order *M*	4
FFT length $N_{\mathrm{fft}}$	256
Cyclic prefix duration $N_{\mathrm{cp}}$	32 samples
Number of employed subcarriers $N_{\mathrm{sc}}$	63
Sampling frequency $F_{s}$	100 MHz

**Table 5:** QAM order *M* for LED1 and LED2.

QAM Order *M*	LED1	LED2
4096	-	from 1 to 9
2048	-	from 10 to 14
1024	from 1 to 6	from 15 to 16
512	from 7 to 10	from 17 to 20
256	from 11 to 13	from 21 to 29
128	from 14 to 15	from 30 to 34
64	from 16 to 18	from 35 to 40
32	from 19 to 23	from 41 to 51
16	from 24 to 27	from 52 to 57
4	from 28 to 39	from 58 to 63

**Table 6:** Measured/simulated throughput (Mbps) of received OFDM signal at different distances *d* and angles ϕ and δ, and with θ=0°.

δ (°)	ϕ = 0°	5°	10°	15°	20°	25°
	*d* = 153.5 cm	154.1 cm	155.9 cm	158.9 cm	163.4 cm	169.4 cm
0°	113.6/113.5	116.6/116.9	122.4/121.0	115.9/112.6	103.2/100.8	91.2/83.6
45°		113.7/117.1	119.4/120.9	112.5/113.6	101.1/101.7	87.1/85.0
90°		116.0/117.2	120.4/121.2	113.6/112.0	101.9/100.3	87.8/83.4
135°		118.7/116.3	118.8/123.1	114.1/111.6	99.8/100.5	87.2/84.3
180°		115.1/117.7	117.4/121.6	112.9/112.4	101.6/100.7	84.8/83.8
225°		117.3/118.6	117.7/122.7	112.6/112.3	102.6/99.3	86.8/84.0
270°		117.9/118.7	120.8/122.1	112.7/113.1	101.6/101.1	89.6/84.6
315°		117.0/117.5	121.0/122.1	113.9/113.4	100.2/100.1	88.5/83.6

**Table 7:** Measured/simulated EVM and throughput of received CAP signal at different distances *d* and angles ϕ, with δ=0° and θ=0°.

ϕ (°)	0°	5°	10°	15°	20°	25°
*d* (cm)	153.5 cm	154.1 cm	155.9 cm	158.9 cm	163.4 cm	169.4 cm
EVM (%)	4.9/5.4	4.8/5.3	4.7/5.1	5.3/5.4	6.0/6.2	7.4/7.6
Thr (Mbps)	87.5/87.5	87.5/87.5	87.5/87.5	87.5/87.5	75.0/75.0	75.0/75.0

## Data Availability

The data presented in this study are available on request from the corresponding author.
